# MicroRNA-146b Promotes Myogenic Differentiation and Modulates Multiple Gene Targets in Muscle Cells

**DOI:** 10.1371/journal.pone.0100657

**Published:** 2014-06-23

**Authors:** Nidhi Khanna, Yejing Ge, Jie Chen

**Affiliations:** Department of Cell and Developmental Biology, University of Illinois at Urbana-Champaign, Urbana, Illinois, United States of America; University of Massachusetts Medical, United States of America

## Abstract

MicroRNAs are established as crucial modulators of skeletal myogenesis, but our knowledge about their identity and targets remains limited. In this study, we have identified microRNA-146b (miR-146b) as a novel regulator of skeletal myoblast differentiation. Following up on a previous microRNA profiling study, we establish that the expression of miR-146b is up-regulated during myoblast differentiation in vitro and muscle regeneration in vivo. Inhibition of miR-146b led to reduced myoblast differentiation, whereas overexpression of miR-146b enhanced differentiation. Computational prediction combined with gene expression information has revealed candidates for miR-146b targets in muscles. Among them, the expression of Smad4, Notch1, and Hmga2 are significantly suppressed by miR-146b overexpression in myocytes. In addition, expression levels of Smad4, Notch1 and Hmga2 are decreased during myoblast differentiation and muscle regeneration, inversely correlating to the levels of miR-146b. Importantly, inhibition of endogenous miR-146b prevents the down-regulation of Smad4, Notch1 and Hmga2 during differentiation. Furthermore, miR-146b directly targets the microRNA response elements (MREs) in the 3′UTR of those genes as assessed by reporter assays. Reporters with the seed regions of MREs mutated are insensitive to miR-146b, further confirming the specificity of targeting. In conclusion, miR-146b is a positive regulator of myogenic differentiation, possibly acting through multiple targets.

## Introduction

Skeletal myogenesis is a highly coordinated process involving myogenic lineage commitment, myoblast proliferation, differentiation and fusion. Myoblasts must undergo a complex series of molecular and morphological changes during this process, the exact mechanism of which is not completely understood. The life-long action of skeletal muscle relies on maintenance and regeneration of myofibers. Muscle repair is carried out by adult stem cells such as satellite cells present between plasma membrane and surrounding basal lamina of mature muscle fibers [1]. Following injury, mitotically quiescent satellite cells re-enter cell cycle, divide and ultimately fuse with existing myofibers or with each other to promote repair and regeneration [2].

MicroRNAs (MiRNAs) are a class of small non-coding RNAs that have emerged as important modulators of gene expression [3]. There are more than 2500 miRNAs in humans (miRBase.org) and they are predicted to target ∼30–40% genes of the human genome. MiRNAs are involved in the regulation of many cellular and developmental processes as diverse as cell proliferation, cell survival, embryonic development and tissue differentiation [4], [5]. Every aspect of skeletal myogenesis has been shown to be regulated by miRNAs [6]. The activity of the miRNA processing enzyme, Dicer, is essential for normal muscle development during embryogenesis. Muscle-specific Dicer knockout mice have severely reduced muscle mass along with abnormal myofiber morphology leading to death within minutes of birth [7]. Various miRNAs have been shown to regulate key steps of skeletal myogenesis, of which the best-characterized myogenic miRNAs are miR-1, 206 and 133 [8]–[10]. To date, 20 or so miRNAs have been reported to regulate myogenesis [11]. Considering the prevalence of miRNA regulation in all aspects of biology, it is likely that additional myogenic miRNAs are to be identified. Indeed, expression profiling has revealed many miRNAs with differential expression patterns during myogenic differentiation [12], and they are likely candidates for novel myogenic regulators.

MiR-146b is conserved among most vertebrates, and its expression increases during mouse prenatal development from E9.5 to E11.5 [13]. The function of miR-146b has been implicated in breast cancer metastasis [14], innate immunity [15], [16], inflammation [17], senescence [18], and glioma cell migration and invasion [19]. MiR-146b is also among the miRNAs identified in microarray studies to be up-regulated during satellite cell activation [20] and myoblast differentiation [12], but a role of miR-146b in skeletal myogenesis has never been reported. In the current study, we examined the potential function of miR-146b in myoblast differentiation.

## Materials and Methods

### Ethics Statement

All animal experiments in this study were performed following protocols approved by the Animal Care and Use Committee at the University of Illinois at Urbana-Champaign, and conforming to the National Institutes of Health standards.

### Antibodies and other Reagents

Anti-MHC (MF20) and anti-myogenin (F5D) were obtained from the Developmental Studies Hybridoma Bank developed under the auspices of the NICHD, National Institutes of Health and maintained by The University of Iowa, Department of Biological Sciences. Anti-tubulin was from Abcam. Antibodies against Hmga2, Smad4 and Notch1 were from Cell Signaling Technology. All secondary antibodies were obtained from Jackson ImmunoResearch Laboratories, Inc. All reagents were from Sigma-Aldrich.

### Cell Culture and Transfection

C2C12 myoblasts were maintained in Dulbecco’s modified Eagle’s medium (DMEM) containing 1 g/L glucose with 10% fetal bovine serum at 37°C with 7.5% CO_2_. Primary myoblasts were maintained in F-10 medium supplemented with 25 ng/ml bFGF and 20% fetal bovine serum at 37°C with 7.5% CO_2_. To induce differentiation, cells were plated on tissue culture plates coated with 0.2% gelatin and grown to 100% confluence for C2C12 and 60–70% confluence for primary myoblasts, changed into differentiation medium (DMEM containing 2% horse serum), and replenished with fresh medium daily for 3 days for C2C12 cells and 2 days for primary myoblasts. HEK293 cells were maintained in DMEM containing 4.5 g/L glucose with 10% fetal bovine serum at 37°C with 5.5% CO_2_. Transfections were performed using Lipofectamine 2000 (Invitrogen).

### Mouse Primary Myoblast Isolation

Primary myoblast isolation was performed as described previously [21]. Briefly, hind limb muscles from 5 to 7-day-old FVB mice were isolated and minced in HBSS, digested in dispase II (2.4 U/mL, Roche) and collagenase D (1.5 U/mL, Roche) solution containing 2.5 mM CaCl_2_ at 37°C for 2 hr. Upon sequential filtering through 70 µm and 40 µm cell strainers (BD biosciences), the cells were collected by centrifugation at 350 g, and resuspended in F-10 culture medium. Serial plating was performed to enrich for myoblasts and eliminate fibroblasts.

### Mouse Muscle Injury and Regeneration

Eight to 10-week-old male FVB mice were used in all the regeneration experiments. Muscle injury was induced by injection of barium chloride (BaCl_2_, 50 µL of 1.2% w/v in saline) into TA muscles as previously described [22]. On various days after injury, the mice were euthanized and the TA muscles were collected, followed by RNA isolation.

### Plasmids and Oligonucleotides

All the reporters were generated by inserting synthetic oligonucleotide DNA linkers of MRE sequences or their mutants into the pMIR-REPORTER vector (Applied Biosystems) downstream of luciferase gene through Hind III and Spe I sites. Native RNA duplexes for miR-146b and siEGFP (siRNA against EGFP) were custom-synthesized by Integrated DNA Technology. miRIDIAN miR-146b mimic and a negative control (cel-miR-67, which has no sequence identity with miRNAs in human, mouse and rat) were purchased from Dharmacon. Locked nucleic acid (LNA) anti-sense oligonucleotides were purchased from Exiqon, Inc.

### Western Blotting

Cells were lysed in a buffer containing 50 mM Tris-HCl, pH 7.2, 150 mM NaCl, 1% NP-40, and 1% protease inhibitor cocktail (Sigma). The lysates were cleared by micro-centrifugation at 13000 rpm, and then mixed with SDS sample buffer. Proteins were resolved on SDS-PAGE and transferred onto PVDF membrane (Millipore), and incubated with various antibodies following the manufacturer’s recommendations. Detection of horseradish peroxidase-conjugated secondary antibodies was performed with Western Lightning Chemiluminescence Reagent Plus (Perkin Elmer Life Sciences, Inc.), and images were developed on x-ray films.

### Immunofluorescence Microscopy and Quantitative Analysis of Myocytes

C2C12 cells differentiated in 12-well plates were fixed and stained for MHC and DAPI as previously described [21]. The stained cells were examined under a Leica DMI 4000B microscope with a 10x dry objective (Leica Fluotar, numerical aperture 0.4), and the fluorescent images were captured at 8-bit at room temperature using a RETIGA EXi camera equipped with Qcapture Pro51 software (QImaging). The images were then pseudo-colored in Adobe Photoshop CS5, where brightness and contrast were adjusted. Fusion index was calculated as the percentage of nuclei in MHC-positive myotubes with ≥2 nuclei. Each data point was generated from randomly chosen microscopic fields containing in total 200 or more nuclei.

### Quantitative Reverse Transcription PCR (qRT-PCR)

Mouse TA muscles were isolated, ground into powder in liquid nitrogen, and lysed in Trizol (Invitrogen). C2C12 cells or mouse primary myoblasts were lysed directly in Trizol. RNA was isolated following the manufacturer’s protocol. Real-time PCR reactions were performed for Smad4, Notch1 and Hmga2 using Syber mix on a StepOnePlus system (Applied Biosystems). β-Actin was used as a reference to obtain the relative fold change for target samples using the comparative CT method. The sequences of PCR primers are as follows. Smad4 forward: AGCCATAGTGAAGGACTGTTGCAG, Smad4 reverse: TACTTCCAGTCCAGGTGGTAGTGC; Notch1 forward: CACCTGTGACCTGCTCA CTC, Notch 1 reverse: ATTGGCACAGGGGTTGG A; Hmga2 forward: GTGCCACAGAAGC GAGGAC, Hmga2 reverse: GCTGCTTTAGAGGGGCTCTT. Mature miR-146b levels were quantified using a qPCR-based Taqman assay kit (Applied Biosystems). SnoRNA-202 was used as the internal control for normalization.

### Luciferase Reporter Assays

HEK293 or C2C12 cells transfected with the luciferase reporters were lysed in Passive Lysis Buffer (Promega), and luciferase assays were performed using the Luciferase Assay Systems kit (Promega) following the manufacturer’s protocol.

### Statistical Analysis

All quantitative data are presented as mean ± standard deviation (SD). Whenever necessary, statistical significance of the data was analyzed by performing one-sample or paired t-tests. The specific types of tests and the *P* values, when applicable, are indicated in figure legends.

## Results

### MiR-146b Expression is Up-regulated during Myogenesis

**Figure 1:**
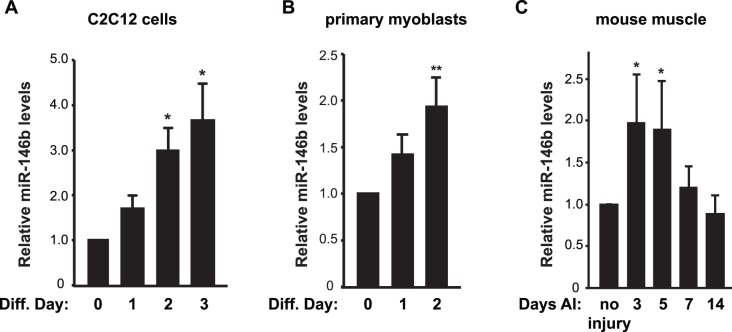
miR-146b expression is up-regulated during myogenesis. (A, B) C2C12 myoblasts (A) and mouse primary myoblasts (B) were induced to differentiate. Total RNA was isolated from the differentiating cells on various days as indicated (diff. day) and subjected to analysis by qRT-PCR to determine the relative levels of mature miR-146b with that on day 0 as 1. Data shown are mean ± SD from three to four independent experiments. (C) Regeneration of mouse TA muscles was induced by BaCl_2_ injury. On various days after injury (AI), total RNA was isolated from the TA muscles and subjected to analysis by qRT-PCR to determine the relative levels of mature miR-146b. Saline injection into contralateral TA muscles served as “no injury” control and was designated as 1. Data shown are mean ± SD with at least three mice per time point. One-sample *t* test was performed to analyze each data point. *P<0.05; **P<0.01.

Our previous microarray profiling of miRNA expression revealed multiple miRNAs that were differentially expressed in differentiated versus undifferentiated mouse C2C12 myoblasts, and miR-146b-5p was among those up-regulated upon differentiation [12]. Dicing of pre-miR-146b stem loop gives rise to two distinct mature miRNA species, with miR-146-5p being the major and miR-146b-3p (or miR-146b*) being the minor one (miRBase.org). For simplicity, we refer to miR-146b-5p as miR-146b from here on. Of note, all reagents used in this study were specific for miR-146b-5p. To validate the microarray data and further examine miR-146b expression, we performed qRT-PCR experiments with RNAs isolated from C2C12 cells over the course of differentiation induced by serum withdrawal. As shown in Fig. 1A, miR-146b levels increased steadily during differentiation and reached ∼3.5-fold by day 3. This expression pattern was also observed during primary myoblast differentiation, albeit to a more modest degree (Fig. 1B).

We also examined miR-146b expression in vivo during muscle regeneration in a mouse model. BaCl_2_ was injected into tibialis anterior (TA) muscle to induce degeneration, followed by myofiber regeneration [22]. As shown in Fig. 1C, expression of miR-146b increased during day 3–5 after injury, a period of satellite cell activation and new myofiber formation, and returned to basal level after that. Taken together, these observations imply that miR-146b may have a positive role in myogenesis.

### MiR-146b Positively Regulates Myoblast Differentiation

**Figure 2:**
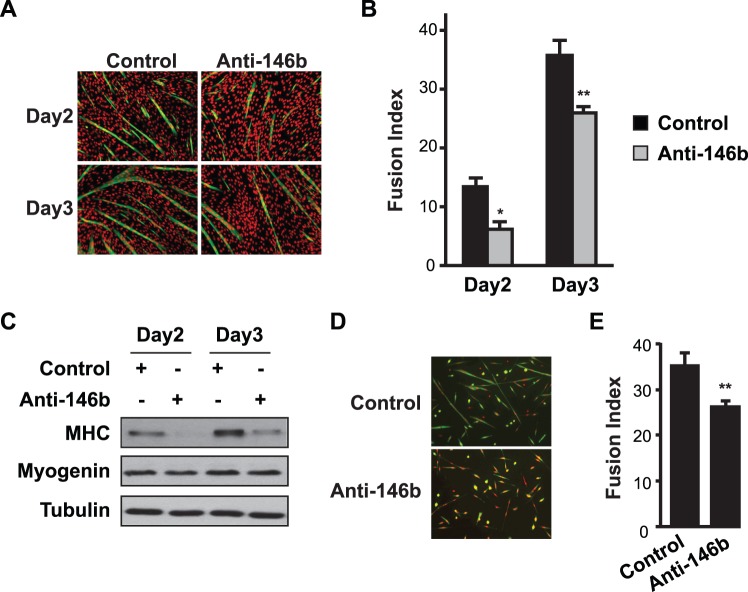
Inhibition of miR-146b suppresses myoblast differentiation. C2C12 myoblasts (A–C) or primary myoblasts (D–E) were transfected with 50 nM LNA-anti-miR-146b for 1 day and then induced to differentiate for 3 or 2 days, respectively. An LNA oligonucleotide with scrambled sequence served as a negative control. (A, D) The differentiated cells were fixed and immunostained for MHC (green) and DAPI (red). (B, E) Fusion indexes were quantified. (C) Cell lysates were subjected to Western blot analysis. In A, C, and D representative results of at least three independent experiments are shown. Data in B & E are shown as mean ± SD from three to four independent experiments. Paired *t* tests were performed to compare the data. *P<0.05; **P<0.01.

To examine a possible role of miR-146b in myoblast differentiation, we inhibited miR-146b function in cells by delivering an anti-sense LNA-oligo by transfection, at an efficiency of ∼75% as previously described [21]. A scrambled LNA oligo with no sequence homology to any known miRNA was used as a control. As shown in Fig. 2A, transfection of LNA-anti-miR-146b into C2C12 cells led to inhibition of myotube formation. Quantification of myotubes revealed significant reduction in fusion index (Fig. 2B). Anti-miR-146b also inhibited the expression of myosin heavy chain (MHC), a late marker of differentiation, with no significant effect on the early differentiation marker myogenin (Fig. 2C). We also examined the effect of miR-146b inhibition on primary myoblast differentiation, and found that anti-miR-146b suppressed differentiation as indicated by impaired myotube formation (Fig. 2D) and reduced fusion index (Fig. 2E). Thus, miR-146b appears to be necessary for optimal myoblast differentiation.

**Figure 3:**
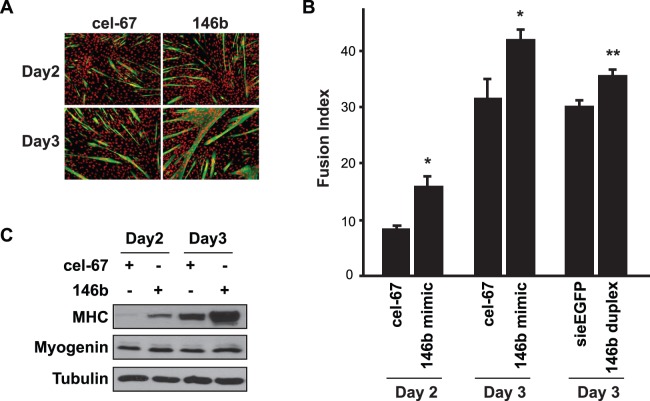
Overexpression of miR-146b promotes myoblast differentiation. (A) C2C12 myoblasts were transfected with 50 nM miRIDIAN miR-146b mimic for 1 day and then induced to differentiate for 3 days. A *C elegans* miRNA (cel-67) mimic was used as negative control. The differentiated cells were fixed and immunostained for MHC (green) and DAPI (red). (B) Fusion indexes for the cells described in A were quantified. In addition, cells transfected with 50 nM native miR-146b duplex, with an siRNA against EGFP (siEGFP) as control, were differentiated and quantified for fusion index. (C) Cells described in A were lysed and subjected to Western blot analysis. In A and C, representative results of at least three independent experiments are shown. Data in B is the mean ± SD from three independent experiments. Paired *t* test was performed to compare the data. *P<0.05.

To further validate this positive function of miR-146b in myoblast differentiation, we introduced a chemically stabilized RNA duplex (miRIDIAN) of miR-146b into C2C12 myoblasts by transfection at ∼90% efficiency as previously reported [21]. The *C elegans* miR-67 (cel-67), with no homology to any known mouse miRNA, was used as a control. Delivery of the stabilized miR-146b into myoblasts (equivalent of overexpressing miR-146b) led to enhanced myotube formation (Fig. 3A), elevated fusion index (Fig. 3B) as well as an increase in MHC expression, but no effect on myogenin expression (Fig. 3C). A native (unmodified) miR-146b duplex also increased fusion index (Fig. 3B), albeit to a lesser degree compared to the stabilized mimic. It is important to note that the passenger strand in the miR-146b miRIDIAN mimic, which was similar (but not identical) to miR-146b*, was chemically modified to prevent its incorporation into the RNA-induced silencing complex (RISC). The fact that the native duplex was less effective than the mimic further confirmed that the passenger strand (miR-146b*-like) was not responsible for the observed phenotype. Taken together, these data strongly suggest that miR-146b is a positive regulator of myoblast differentiation.

### Smad4, Hmga2 and Notch1 are Targets of miR-146b during Myoblast Differentiation

**Figure 4:**
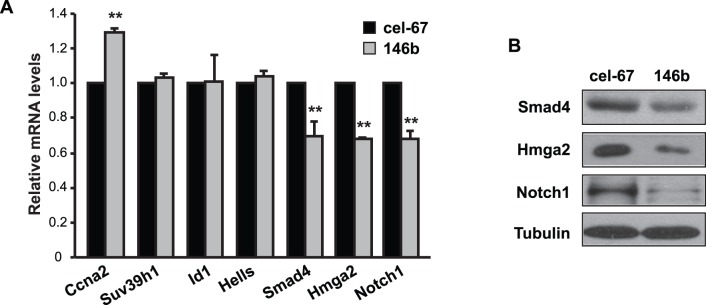
Overexpression of miR-146b suppresses expression of Smad4, Hmga2 and Notch1 in myoblasts. (A) C2C12 myoblasts were transfected with 50 nM miRIDIAN miR-146b mimic or cel-miR-67 mimic as control. After 24 hours, cells were lysed for RNA isolation, followed by qRT-PCR to measure mRNA levels for the genes shown. Relative mRNA levels are shown with that of cel-miR-67 as 1. Data shown are the mean ± SD from three independent experiments. One-sample *t* test was performed. **P<0.01. (B) Cells as described in A were lysed after 48 hours of transfection and subjected to Western blotting analysis. Representative results of three independent experiments are shown.

MiRNAs modulate gene expression by targeting mRNAs for translational repression and mRNA degradation. In most cases, miRNAs bind to their target mRNAs in the 3′UTR by imperfect base pairing. Perfect and contiguous base pairing of mature miRNA nucleotides 2 to 8 (seed region) to its target mRNA has been found to be critical for the majority of mRNA targeting [3], although seedless targeting is also reported [23]–[25]. Computational target prediction by miRanda, TargetScan and Pictar altogether yielded hundreds of putative targets for miR-146b. Because miRNAs almost always trigger the decay of their mRNA targets [26], [27], within the predicted miR-146b target list we looked for genes that were reported to be down-regulated at the mRNA levels during differentiation of C2C12 cells as well as implied in myogenesis. We then examined the effect of miR-146b overexpression on the expression of each of those genes in C2C12 cells. Smad4, Hmga2 (high-mobility-group proteins containing AT-hook DNA binding domains), and Notch1 emerged as strong candidates for miR-146b targets because the expression of each was dampened by miR-146b overexpression (Fig. 4A). The modest degree of reduction (20–30%) in the mRNA levels is commonly observed for target genes in response to the overexpression of a single miRNA. Examples of genes not suppressed by miR-146b overexpression are also shown in Fig. 4A, including Ccna2, Suv39h1, Id1, and Hells. The increase of Ccna2 levels is likely an indirect effect of miR-146b overexpression. Importantly, the protein levels of Smad4, Hmga2, and Notch1 were also reduced by the overexpression of miR-146b in myoblasts (Fig. 4B).

**Figure 5:**
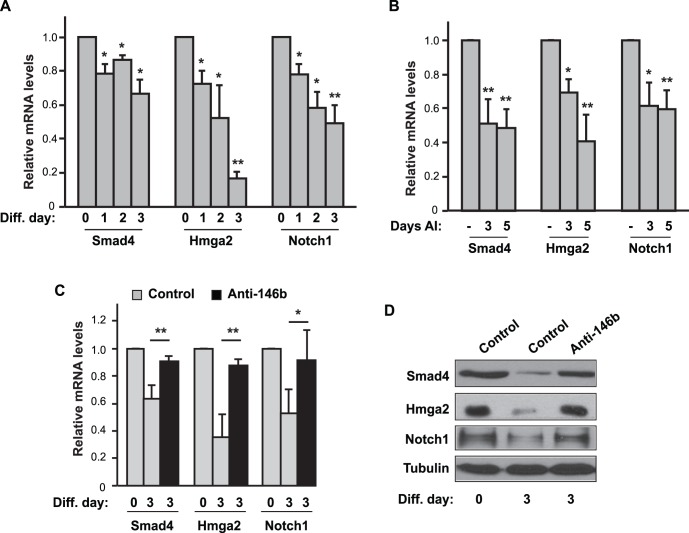
Smad4, Hmga2 and Notch1 are targets of miR-146b during myoblast differentiation. (A) C2C12 cells were induced to differentiate, and total RNA was isolated from the differentiating cells on various days as indicated (Diff. day) and subjected to analysis by qRT-PCR to determine the relative levels of mRNA for each gene, with that at day 0 as 1. (B) Regeneration of mouse TA muscles was induced by BaCl_2_ injury. On day 3 and day 5 AI, the TA muscles were isolated for RNA extraction, followed by qRT-PCR assays to measure relative mRNA levels. Saline injection into contralateral TA muscles served as control (“−”) and was designated as 1. (C) C2C12 cells were transfected with 50 nM LNA-anti-miR-146b or LNA control for 1 day and then induced to differentiate for 3 days. Total RNA was isolated on day 0 and day 3 of differentiation and analyzed by qRT-PCR. (D) Cells as described in C were lysed and subjected to Western blotting analysis. Data shown are mean ± SD from three to four independent experiments (A & C) or at least three mice per time point (B), or representative results of three independent experiments (D). One-sample *t* test was performed to analyze data in A & B, and paired t test was performed for data in C. *P<0.05; **P<0.01.

Protein levels for each of the putative targets of miR-146b have been reported to be down-regulated during myoblast differentiation [28]–[30] (also see Fig. 5D), inversely correlating with the increased expression of endogenous miR-146b during myoblast differentiation (Fig. 1A&B). Of the three genes, Smad4 had previously been reported to be a miR-146b target in immune cells [16], [31], but its regulation in myoblasts by miR-146b has never been examined. To gain further insight into the regulation of Smad4, Hmga2, and Notch1 during myoblast differentiation, we measured mRNA levels for each of these genes throughout the course of differentiation. As shown in Fig. 5A, a significant reduction in mRNA expression levels was observed for all three genes upon differentiation. Next, we examined the expression of these genes during muscle regeneration. We reasoned that the targets of miR-146b in regenerating muscles would be down-regulated upon miR-146b up-regulation. Indeed, we found that the mRNA levels of Smad4, Notch1 and Hmga2 were all reduced by 30–60% on day 3 and day 5 after injury (Fig. 5B). These expression patterns are perfectly in line with the possibility that miR-146b targets these genes both in vitro and in vivo to impact skeletal myogenesis. Importantly, inhibition of endogenous miR-146b by the antisense LNA oligo almost completely prevented the decline in both mRNA and protein expression of Smad4, Hmga2, and Notch1 during myoblast differentiation (Fig. 5C&D), further supporting a critical role of miR-146b in suppressing those genes.

### MiR-146b Directly Targets the MREs in 3′UTRs of Smad4, Notch1, and Hmga2

**Figure 6:**
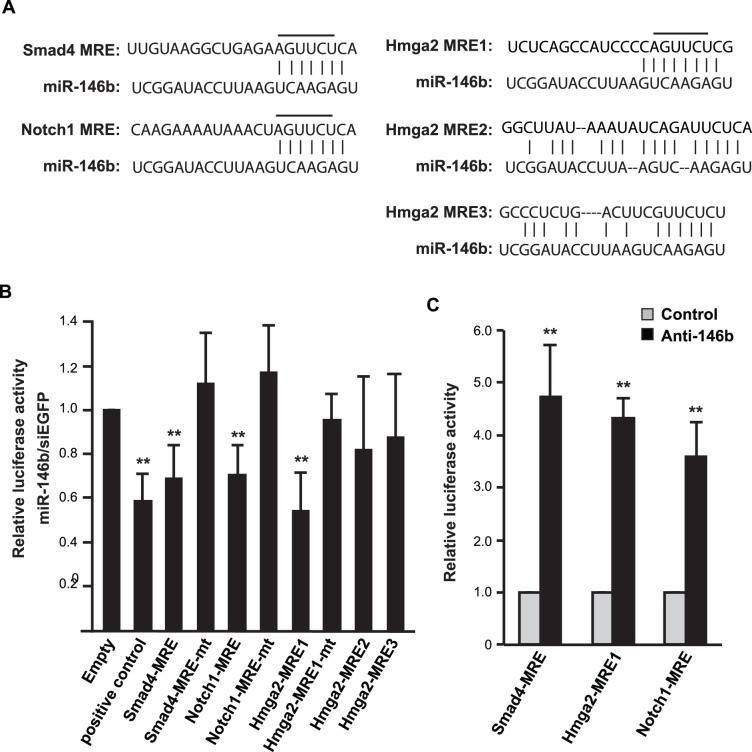
miR-146b directly targets the MREs in 3′UTRs of Smad4, Hmga2 and Notch1. (A) Predicted miR-146b target sites in the 3′UTRs of mouse Smad4, Notch1 and Hmga2 are shown. The nucleotides in the seed region that were changed to complementary sequence in the mutant 3′UTR reporters are indicated by lines above them. (B) The MRE reporters or their mutant counterparts were cotransfected with miR-146b duplex into HEK293 cells, with siEGFP as a negative control. Twenty-four hours after transfection the cells were lysed and subjected to luciferase assays. A sequence perfectly complementary to the active strand of miR-146b was cloned into the reporter and used as a positive control. (C) The MRE reporters were transfected into C2C12 cells together with 50 nM LNA-anti-miR-146b or LNA control for 1 day, followed by cell lysis and luciferase assays. All data shown are mean ± SD from three independent experiments. One-sample *t* test was performed to analyze each data in B&C. **P<0.01.

3′UTRs of Smad4 and Notch1 are each predicted to have a single miRNA recognition element (MRE) for miR-146b, which is broadly conserved in vertebrates. Hmga2, on the other hand, contains 3 predicted MREs for miR-146b in its 3′UTR (Fig. 6A). To assess whether miR-146b directly regulates one or more of these 3′UTRs, we constructed reporters containing one copy of each putative MRE downstream of the luciferase gene. These reporters were then transfected in HEK293 cells, a non-myogenic cell line with little endogenous miR-146b (data not shown), along with the miR-146b duplex. As shown in Fig. 6B, miR-146b targeted the Smad4 and Notch1 MREs, as indicated by the repression of reporter activities to a similar degree as its suppression of a positive control reporter containing sequences perfectly complementary to miR-146b. For Hmga2, miR-146b targeting was limited to only one of the three predicted MREs (Fig. 6B). To further validate the specificity of the targeting, we constructed reporters with the seed regions mutated in the MREs (Fig. 6A). These mutant reporters were completely resistant to the presence of miR-146b duplex (Fig. 6B), confirming the specificity of the miR-146b action. To examine this targeting in a more physiologically relevant context, we expressed the MRE reporters in C2C12 cells, and introduced LNA-anti-miR-146b. As shown in Fig. 6C, inhibition of endogenous miR-146b was sufficient to activate the reporters, suggesting that miR-146b normally suppresses those MREs in myocytes.

## Discussion

Our results have identified miR-146b as a novel regulator of myoblast differentiation. In addition, we provide evidence to establish Smad4, Notch1 and Hmga2 as direct targets of miR-146b during myoblast differentiation. Expression of miR-146b is up-regulated during myoblast differentiation and muscle regeneration, accompanied by down-regulation of the target genes. Our previous miRNA profiling results indicated that miR-146b was the fourth most up-regulated miRNA after miR-1, miR-206, and miR-133 upon differentiation of C2C12 cells [12]. While the myogenic roles of the other three miRNAs have been well documented [8]–[10], this is the first report of miR-146b as a myogenic regulator, which also attests to the power of expression profiling in predicting function. New myogenic miRNAs may continue to be discovered by this approach.

Major myogenic miRNAs, miR-1, miR-133, and miR-206, are expressed in muscles under the control of the myogenic transcription factors SRF, MyoD, and MEF2 [32]–[35]. One or more of those transcription factors may also regulate the expression of miR-146b. Another potential regulator is NF-κB, which has been reported to regulate miR-146b biogenesis in immune cells [16]. Future investigations will probe the mechanisms of miR-146b biogenesis in myogenesis.

Transforming growth factor β (TGFβ)/bone morphogenetic protein (BMP) signaling pathways regulate satellite cell activation and proliferation during muscle development [36]. TGFβ and BMP signal through specific Smad proteins, which on activation form a complex with the common regulator Smad4 to regulate gene expression. Similarly, Notch signaling is known to control satellite cell quiescence and activation [29], [37]–[39]. In addition to regulating myoblast proliferation, these signaling pathways inhibit the transcriptional activity of myogenic regulatory factors (MRFs) and prevent myoblast differentiation, maintaining muscle stem cell self-renewal [38], [40], [41]. Hmga2 is also a key regulator of satellite cell activation and proliferation both in vivo and in vitro [30]. As cell cycle withdrawal is a prerequisite for myogenic differentiation, these regulators of myoblast proliferation need to be down regulated upon entering myogenic differentiation. In fact, forced expression of either Hmga2 or Smad4, or constitutive activation of Notch, is sufficient to prevent myoblast differentiation [28], [30], [42]. Here we provide compelling evidence for miR-146b regulation of these inhibitors of myogenesis. We propose that the increased level of miR-146b during myogenesis serves to posttranscriptionally suppress Smad4, Notch1, and Hmga2 (and potentially other genes) in order to allow the activation of myogenic differentiation program.

Smad4 has been shown to be targeted by miR-146b in human papillary thyroid cells on the same MRE as that on the mouse gene discovered in our study [31], suggesting that this regulation may exist in multiple cell/tissue types. There are four mammalian Notch receptors, Notch1–4, of which Notch1 and 3 are known to be anti-myogenic [43], [44]. Regulation of Notch3 by myogenic miRNAs, miR-1 and 206, has been reported [44], and now our findings reveal targeting of Notch1 by miR-146b. Hence, a concerted suppression of Notch 1 and Notch 3 can be achieved, as miR-1, miR-206, and miR-146b are all up-regulated upon differentiation [12].

It is now commonly accepted that the regulation of any gene is rarely controlled by a single miRNA. Rather, multiple miRNAs often coordinate to modulate the expression of a gene [45]. It is unlikely that miR-146b is solely responsible for suppressing the target genes we have identified during myogenic differentiation. In fact, miR-26a has been reported to target Smad4 during myogenesis [28] and it is conceivable that miR-26a and miR-146b act together to regulate Smad4. Similarly, Hmga2 has been reported to be targeted by let-7 [46] and miR-98 [47] in cancer cells, both of which are up-regulated during myoblast differentiation [48], [49]. Hence, a concerted targeting of Hmga2 by let-7, miR-98 and miR-146b during myoblast differentiation is possible. Regardless of the potential coordination, however, the contribution of miR-146b may be indispensible in bringing the levels of those genes below a threshold for the activation of the myogenic program to occur. This notion is supported by our observation that inhibition of miR-146b almost completely prevents down-regulation of Smad4, Notch1, and Hmga2 (Fig. 5C&D).

MicroRNAs hold the potential as therapeutic targets or tools in aging and dystrophic muscles. For instance, over-expression of miR-1/206 suppresses rhabdomyo-sarcoma development through c-met expression [50]. Additionally, intramuscular injections of miR-1, miR-206, and miR-133 in rat skeletal muscle promote muscle regeneration [51], and so does intramuscular injection of anti-miR-125b, a negative regulator of myogenesis [21]. The physiological significance and therapeutic potential of miR-146b as a myogenic regulator warrants future investigations.

## Supporting Information

Figure S1 **Original Western blot images for** **Figs. 2C** **,** **3C** **,** **4B** **,** **5D** **.**
(TIF)

Table S1 **Original luciferase assay data for** **Fig. 6** **.**
(XLSX)
