# Supplementary figures and images for: MicroRNA-146b Promotes Myogenic Differentiation and Modulates Multiple Gene Targets in Muscle Cells
Source: PLoS One. 2014 Jun 23;9(6):e100657. doi: 10.1371/journal.pone.0100657 (PMC4067360; doi:10.1371/journal.pone.0100657)

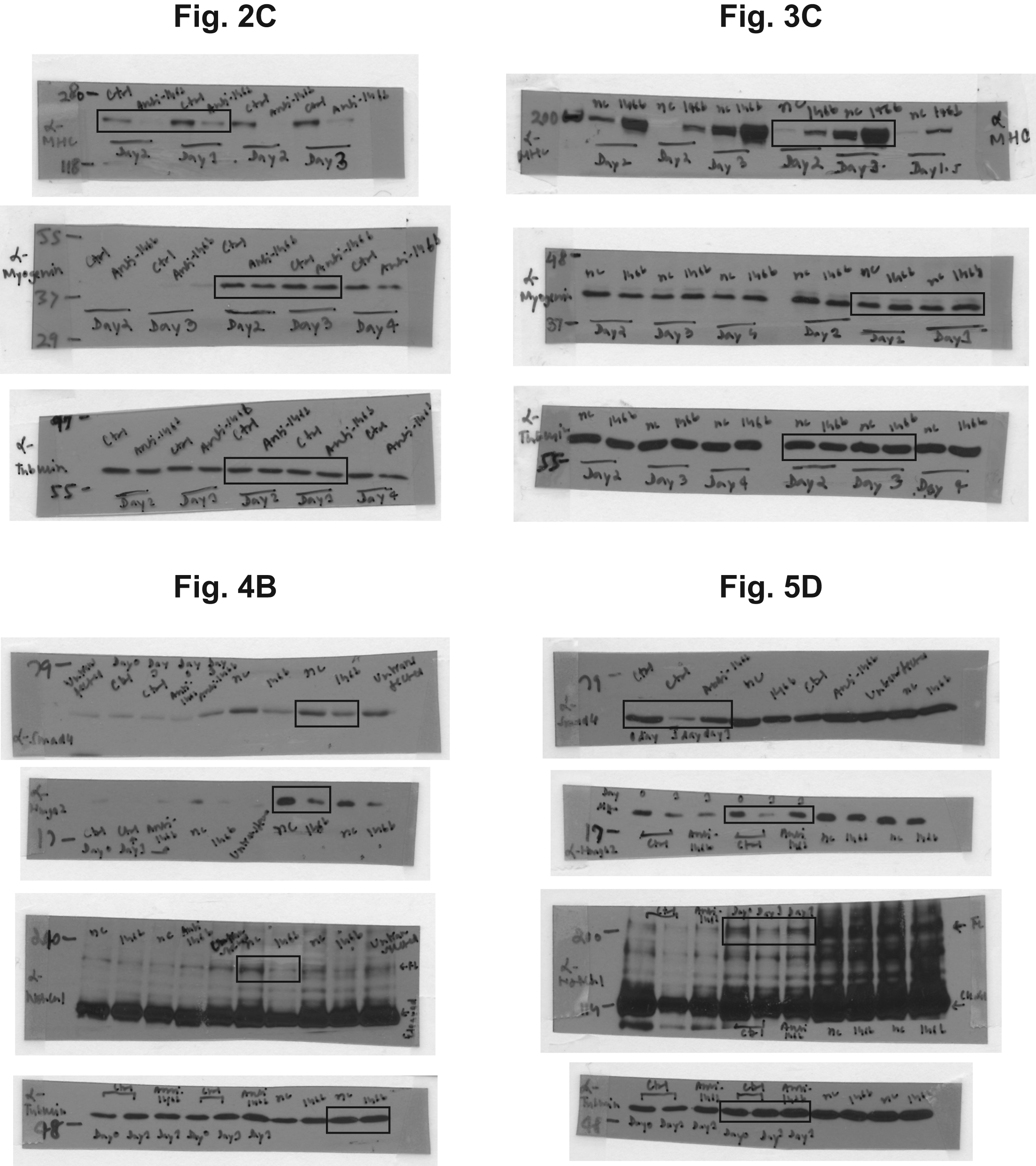

Supplement: Figure S1 — Original Western blot images for Figs. 2C , 3C , 4B , 5D . (TIF) [file pone.0100657.s001.tif]
